# PERSPECTIVES OF UNDER-RESOURCED PATIENTS ABOUT THE MEDICARE ANNUAL WELLNESS VISIT

**DOI:** 10.1093/geroni/igac059.2584

**Published:** 2022-12-20

**Authors:** Christine Ferrone, Roberta Goldman, Joanne Wilkinson, Jeffrey Borkan, Phillip Clark

**Affiliations:** University of Rhode Island, Kingston, Rhode Island, United States; Warren Alpert Medical School of Brown University, Pawtucket, Rhode Island, United States; Alpert Medical School of Brown University, Pawtucket, Rhode Island, United States; The Warren Alpert Medical School of Brown Univerity, Providence, Rhode Island, United States; University of Rhode Island, Kingston, Rhode Island, United States

## Abstract

The Medicare Annual Wellness Visit (AWV) involves a personalized risk assessment and prevention plan to help prevent disease and disability among older adults. Though studies have shown that AWV uptake is suboptimal, little is known about the perspectives and experiences of patients completing it. Family medicine physicians are conducting AWVs at a Rhode Island pilot teaching clinic participating in the GWEP's Age-Friendly Health Systems Initiative. To evaluate perspectives of the clinic's under-resourced patients, 20 qualitative telephone interviews were conducted following their AWV. Interview recordings were analyzed for content and themes. Few patients recognized the term ‘Annual Wellness Visit’ or recognized it was significantly different from other visits: “I noticed it was a bit different, but not that different. I’m usually not covered for a visit like that by Medicare.” Mental health, mobility and medications were discussed at most of the AWVs. Some patients noticed that a physician did the medication review instead of the nurse. Patients had varying experiences of how advance directives were discussed, and some thought it unnecessary since a family member knows their preferences. A few patients were dissatisfied that their physician did not focus on their chronic or acute health problems. However, most patients “trusted” their physicians to do “whatever is necessary” for them, and were glad to have had the AWV because “instead of talking about being sick all the time, talking about how to stay well”. Recommendations for patient education to improve understanding and the experience of the AWV will be offered.

